# Older Age Is Associated with Peripheral Blood Expansion of Naïve B Cells in HIV-Infected Subjects on Antiretroviral Therapy

**DOI:** 10.1371/journal.pone.0107064

**Published:** 2014-09-10

**Authors:** Puja Van Epps, Roy M. Matining, Katherine Tassiopoulos, Donald D. Anthony, Alan Landay, Robert C. Kalayjian, David H. Canaday

**Affiliations:** 1 Geriatric Research Center Clinical Core (GRECC), Department of Infectious Diseases, Louis Stokes Cleveland VA Medical Center, Case Western Reserve University, Cleveland, Ohio, United States of America; 2 Harvard School of Public Health, Boston, Massachusetts, United States of America; 3 Department of Immunology and Microbiology, Rush Medical Center, Chicago, Illinois, United States of America; 4 Department of Infectious Diseases, Metrohealth Medical Center, Cleveland, Ohio, United States of America, and Geriatric Research Center Clinical Core (GRECC), Louis Stokes Cleveland VA Medical Center, Cleveland, Ohio, United States of America; New York University, United States of America

## Abstract

Older HIV infected subjects were previously found to have significant B cell expansion during initial antiretroviral therapy in a prospective age-differentiated cohort of older and younger (≥45 vs. ≤30 years) HIV-infected subjects initiating antiretroviral therapy (ART) through the AIDS Clinical Trials Group. Here to further describe this expansion, using a subset of subjects from the same cohort, we characterized B cell phenotypes at baseline and after 192 weeks of ART in both older and younger HIV-infected groups and compared them to uninfected age-matched controls. We also examined whether phenotypes at baseline associated with response to tetanus and hepatitis A vaccine at 12 weeks. Forty six subjects were analyzed in the HIV infected group (21 older, 25 younger) and 30 in the control group (15 per age group). We observed naïve B cells to normalize in younger subjects after 192 weeks of ART, while in older subjects naïve B cells increased to greater levels than those of controls (p = 0.045). Absolute resting memory (RM) cell count was significantly lower in the older HIV infected group at baseline compared to controls and numbers normalized after 192 weeks of ART (p<0.001). Baseline RM cell count positively correlated with week 12 increase in antibody to tetanus vaccine among both younger and older HIV-infected subjects combined (p = 0.01), but not in controls. The age-associated naïve B cell expansion is a novel finding and we discuss several possible explanations for this observation. Relationship between RM cells at baseline and tetanus responses may lead to insights about the effects of HIV infection on B cell memory function and vaccine responses.

## Introduction

The effects of HIV infection on B lymphocytes include reduced total B lymphocyte counts, hypergammaglobulinaemia and increased risk of B cell malignancies [1]–[3]. Untreated HIV infection is associated with reduced percentages of naive B cells, increased percentages of immature transitional cells, and expansion of memory B cells in the peripheral blood that exhibit increased turnover and greater susceptibility to apoptosis [3]–[5]. Chronic immune activation during HIV infection may contribute to reduced B cell function, particularly within these memory subsets [3].

ART, especially if initiated early in infection, is associated with recovery of total B cell counts and normalization of most B cell subpopulations, but resting memory cell recovery is incomplete [1], [3], [4]. The aging bone marrow has a reduced ability to generate naive B cells, resulting in a diminished capacity for older individuals to respond to neoantigens [6]. During HIV infection, older individuals have a reduced ability to generate naive T cells, but the effects of aging on B cells in the setting of HIV-infection is not known [7], [8]. Pensieroso et al. have previously reported differences in distributions of B cell subsets in a cross-sectional analysis among HIV-infected (treated and untreated) as well as uninfected controls and elite controllers [9]. Using multivariable analysis they investigated whether age was a factor associated with alteration of B-cell subpopulations. Age related differences were not a primary focus of that study and was not found to correlate with any of the B-cell subpopulations.

In a previous study we observed that older HIV infected subjects had a significantly lower restoration of naïve CD4 cells upon initiation of ART, but expansion of total B cell counts, to levels that were significantly higher than those found in age matched HIV-uninfected controls [10]. Within the younger group of HIV-infected subjects B cell counts normalized, but did not expand beyond levels of HIV-uninfected young control subjects. To better understand these observations, using a subset of subjects from this cohort we compared the changes in B cell subset frequencies in response to ART between two age groups, and we examined associations between subsets and the antibody responses to vaccination with tetanus, a recall antigen, and hepA, a neoantigen.

## Methods

### Study Subjects

This research was granted a formal waiver by the Institutional Review Board at Case Western Reserve University. This analysis involves participants with cryopreserved samples available who enrolled into ACTG 5015, a prospective, multicenter study that compared immune and viral responses to antiretroviral therapy (ART) with stavudine, emtricitabine, and lopinavir/ritonavir in ART-naïve older and younger (ages ≥45, and 18–30 years, respectively) HIV-infected subjects over 192 weeks [11]. The original ACTG 5015 cohort included 45 older and 45 younger adults however only the first 55 subjects participated in the immunology sub study (ACTG 5015s) that also included immunologic markers and vaccination with tetanus and hepatitis A. Samples (n = 30) were also included from ACTG 5113, a contemporaneous study of HIV-uninfected healthy controls [11]. The selection of samples for the current study was based on sample availability and viability. Viable samples were available from 46 HIV infected subjects (among all study participants, including immunology sub study participants) and 30 controls. Median ages among the younger groups were 26 and 25 years for HIV-infected and uninfected controls respectively, while the median age was 49 for both the older HIV-infected and uninfected controls. All HIV-infected participants were vaccinated at baseline with tetanus toxoid (Aventis Pasteur, Swiftwater, Pennsylvania; Lederle-Praxis, West Henrietta, New York; or Wyeth-Ayerst, Philadelphia, Pennsylvania, USA), and those without serologic evidence of hepatitis A (n = 24) also received hepA vaccine (Smith-Kline Beecham, King of Prussia, Pennsylvania, USA); the uninfected control subjects were all hepatitis A antibody negative and received both vaccines. All study participants provided written informed consent, and each participating study site received approval from its designated institutional review board. The expansion of total B cells in older adults after 192 weeks of ART and responses to tetanus and hepatitis A vaccines were reported in the larger cohort however B cell phenotype analysis was not done in that study. The B cell phenotype changes in response to ART and the correlation of vaccine responses to B cell phenotypes is a new analysis and being reported in the current study.

Absolute B cell counts were enumerated at the time of the original study using freshly obtained whole blood. B cell phenotypes were measured from cryopreserved PBMCs at weeks 0 and 192 for HIV-infected participants; single time point cryopreserved samples were available for the controls. Phenotypic analysis using flow cytometry was performed immediately after thawing.

### B Lymphocyte Phenotypic Analysis

Surface staining of PBMCs was performed with the following flourochrome conjugated monoclonal antibodies: anti-CD19 Pacific Blue, anti-CD10 APC, anti-CD20 APC-Cy7, anti-CD21 FITC, anti-CD27 PE (all Biolegend). Viability Live/Dead yellow dye (Invitrogen) was added to exclude dead cells from analysis. Whenever enough cells were available, corresponding isotype control (Biolegend) staining was also performed. In the event of insufficient cells to run isotype control internal gating controls using markers that provided the cleanest separation between populations were used. Data were acquired on LSRII flow cytometer (BD Biosciences) and analyzed using FlowJo 7.6.4 software (Tree Star, Ashland, OR). Lymphocytes were identified using forward and side scatter, and further gated to include only singlet events and live cells (Figure S1). CD19+ cells were subsequently gated to determine the following B cell subsets: CD10+CD27− immature transitional (IT), CD10−CD21+CD27− naïve, CD10−CD21+CD27+ resting memory (RM), CD20+CD21−CD27+ mature activated (MA) and CD10−CD21−CD27− tissue like memory (TLM). PBMC samples with less than 50% viability on trypan blue stain at the time of thaw or live/dead staining were discarded. 5×10^5^–10^6^ total events were captured. In addition there had to be at least 1000 CD19+ cells to analyze the B cell subsets.

### Statistical Analysis

The stratified (by sex) Wilcoxon rank-sum test was used to compare B-cell phenotypes between HIV-infected subjects and healthy controls within each age group (at week 0 and 192 for HIV-infected subjects; at week 0 for uninfected controls); shift estimates and 95% confidence intervals (CIs) were obtained by inverting this test. Associations between B cell phenotypes at baseline and the change in antibody from baseline to week 12 were examined by Spearman rank correlations and by linear regression models. These examined interactions between HIV-infection status and age group. An additional analysis stratified for sex and race (black vs. not black) was performed. Statistical significance was defined as p<0.05. No adjustment was made to account for multiple testing in these analyses.

## Results

Forty-six HIV-infected subjects (n = 21 and 25 for older and younger respectively) and 30 age-matched controls (n = 15 each for older and younger) were included in this analysis. Thirty eight of 46 HIV-infected subjects completed 192 weeks of follow up (19 of 21 [90%] older; 19 of 25 [76%] younger; p = 0.26). Baseline characteristics are included in table 1. Compared with the group of HIV-infected subjects, the uninfected healthy control group included significantly more women (p = 0.007) and significantly fewer African-Americans and Latinos (p<0.001). Baseline total CD4 counts were significantly lower in older HIV-infected subjects compared to younger group (p<0.005). Baseline total CD4 counts and naïve CD4 counts were significantly lower in HIV-infected subjects compared to controls (p<0.001 and p = 0.005 respectively). The median log10 viral load (VL) at baseline was not statistically different among the older HIV-infected subjects (5.1 vs. 4.3 log10 copies/mL; p = 0.32). There were no significant age-group differences in the percentage of subjects who achieved initial VL suppression to <200 copies/mL (19 of 21 [90%] older vs. 17 of 21 [81%] younger; p = 0.66). Number of subjects achieving viral suppression after 192 weeks of therapy was not statistically different between the two group (18 of 19 [95%] older vs. 15 of 19 [79%] younger; p = 0.34).

**Table 1:** Baseline characteristics of study participants.

	HIV-infected		Uninfected controls	
	Older	Younger	Older	Younger
N	21	25	15	15
Median age (range)	49 (48,55)	26 (23,28)	49 (46, 53)	25 (23, 28)
Sex n, (% Male)	14 (66.6)	21 (84.0)	7 (46.7)	6 (40.0)
Race n, (% Black)	15 (71.4)	20 (80.0)	2 (13.3)	5 (33.3)
CD4 count cells/mm^3^ (range)	158 (35, 340)	266 (203, 430)	900 (653, 1141)	776 (630, 837)
Naïve CD4 count cells/mm^3^ (range)	54 (11, 76)	101 (78, 175)	295 (226, 491)	369 (288, 417)
B cell count cells/mm^3^ (range)	124 (55, 230)	124 (88, 179)	213 (125, 245)	215 (127, 269)
Log10 HIV-RNA copies/ml (range)	5.1 (4.0, 5.6)	4.3 (4.1, 5.1)	-	-

### Effect of Untreated HIV Infection on B Cell Subtypes

**Figure 1:**
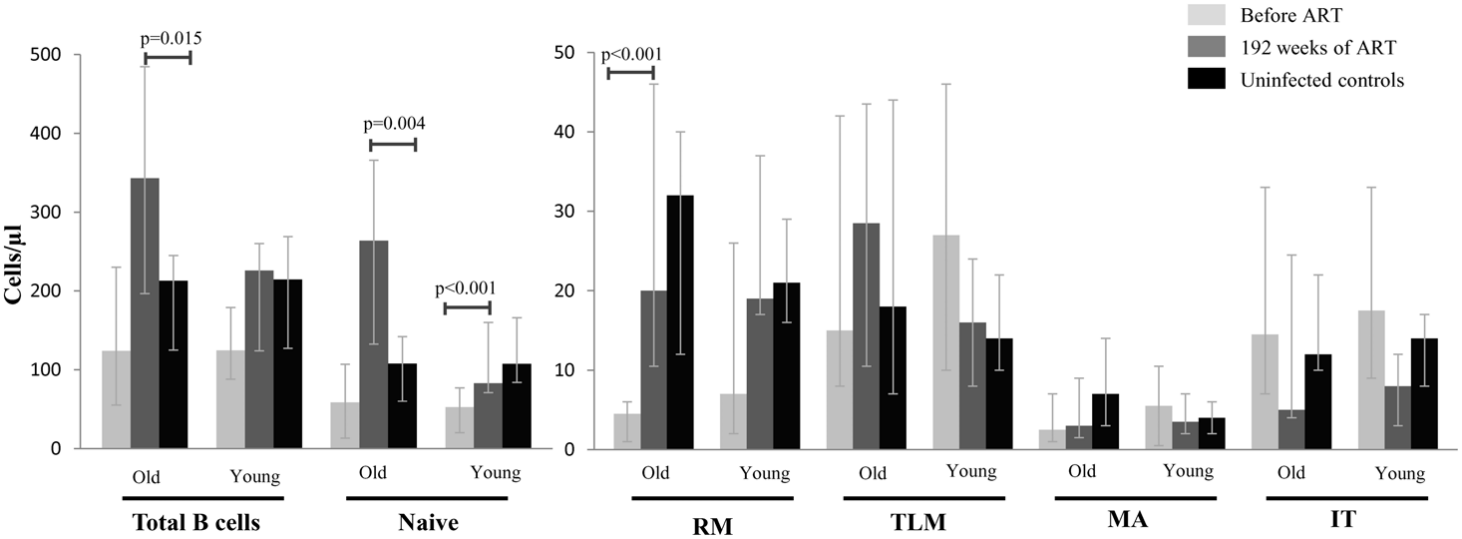
Absolute B cell phenotype counts before and after antiretroviral therapy. Changes in absolute B cell count and B cell subset counts at baseline before ART (light grey) and after 192 weeks of ART (dark grey) compared to their age-matched controls (black) in older and younger groups. Only the significant P-values are marked. Bars represent median; error bars represent interquartile ranges. RM: resting memory, TLM: tissue like memory, MA: mature activated, IT: immature transitional.

**Figure 2:**
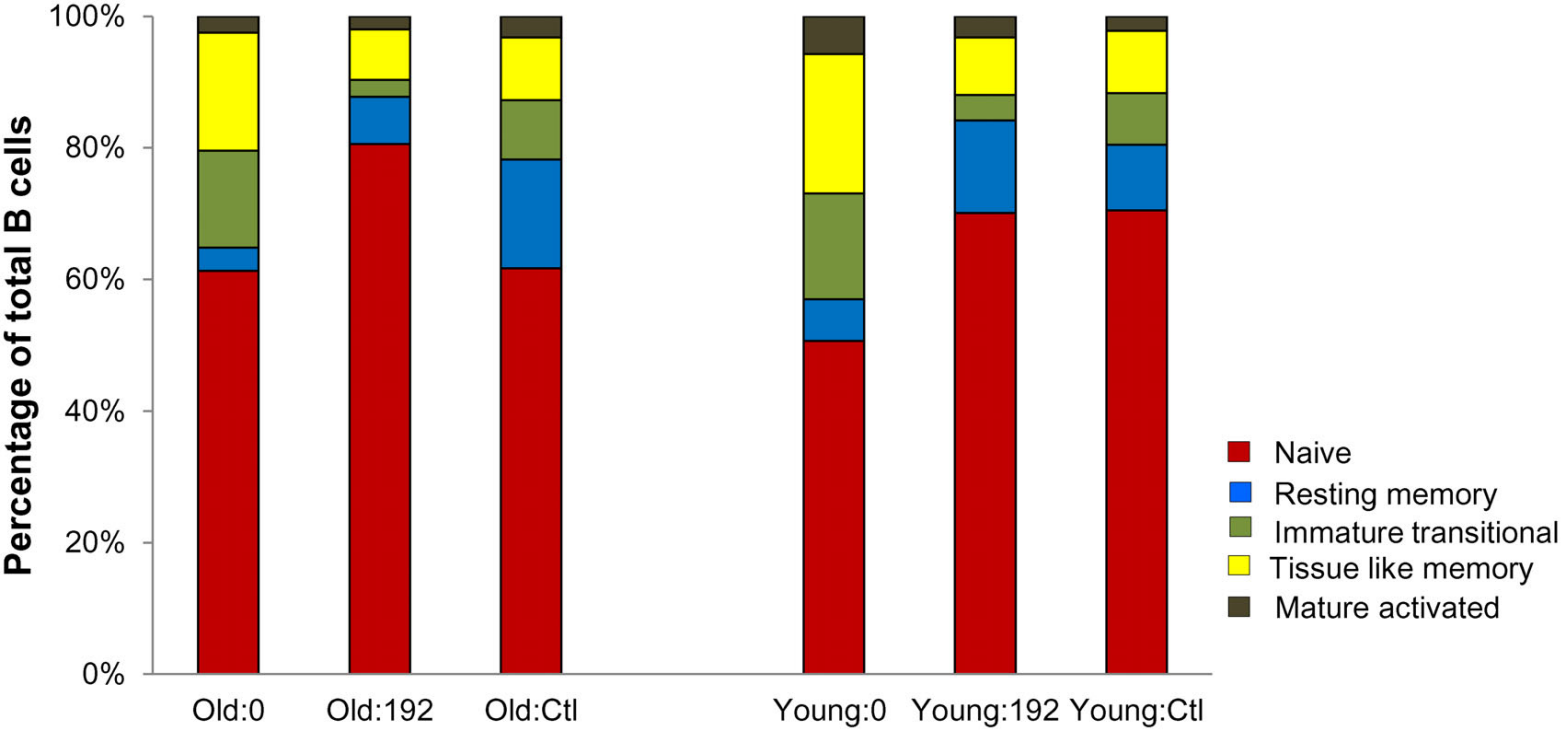
B cell phenotype distribution before and after antiretroviral therapy. Change in phenotype distribution in old and young age groups compared with their respective age-matched controls. 0 and 192 refer to time on ART in weeks; Ctl refer to respective age matched uninfected controls.

While untreated HIV infection was associated with a trend toward lower total B cell count among the younger, group it was not different from uninfected age-matched controls in the older group (p = 0.05 for younger and p = 0.12 older; Figure 1). Untreated infection was associated with significantly lower naïve B cell count in younger, but not in older subjects (p<0.001 and p = 0.11 for younger and older, respectively; Figure 1). In contrast, RM cells were significantly lower in older subjects (p<0.001), but not in younger subjects, compared to controls (p = 0.06; Figure 1). Similarly, the older group had a significantly lower percentage of RM component at baseline compared to the uninfected controls (3% vs. 15%; p<0.001; Figure 2). While the IT, TLM and MA absolute counts did not differ significantly compared to controls in either age-group, there were intergroup differences in percentages of these subtypes. Younger infected subjects had significantly higher percentages of TLM (18% vs. 9%; p = 0.006) and IT cells (14% vs. 7%; p = 0.02), compared to controls (Figure 2). Among older subjects, the percentage of TLM was higher among the HIV infected group compared to controls (17% vs. 9%; p = 0.01), but there were no significant differences in the percentages of IT or MA cells (Figure 2).

### Vaccine Antibody Responses to B cell Phenotypes at Baseline

**Figure 3:**
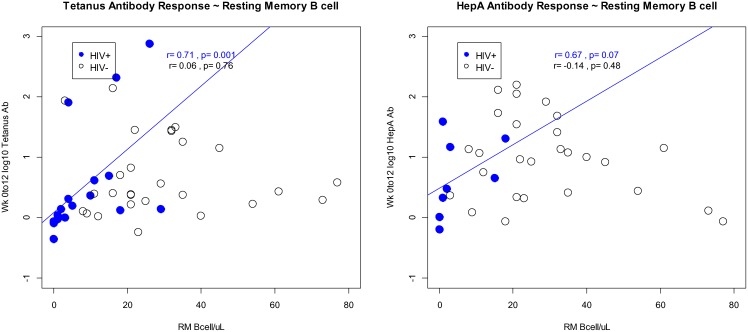
Correlations between antibody responses to vaccines and resting memory count at baseline in HIV infected and uninfected controls. a. Tetanus vaccine b. Hepatitis A vaccine.

Higher increases in week-12 tetanus antibody from baseline were associated with higher baseline RM counts, but this association was only seen in HIV-infected subjects (r = 0.71, p = 0.001for HIV infected subjects; Figure 3). A similar interaction may also be the case for hepA antibody responses, where there was a trend towards higher week-12 antibody increases from baseline associated with higher baseline RM counts among HIV-infected subjects (r = 0.67, p = 0.07 among HIV infected subjects, only; Figure 3). This interaction was not observed among HIV-uninfected controls. Younger subjects also had significantly higher hepA antibody responses to vaccination, regardless of HIV infection status (p = 0.003); there were no significant age group differences in the antibody response to tetanus vaccination. There were no associations between the remainder of B cell phenotypes at baseline and vaccine responses.

### HIV and Age Effects on B Cells after ART

After 192 weeks of ART, the peripheral blood B cell count normalized in younger subjects to levels comparable to those of controls but increased significantly in older subjects to levels above controls (p = 0.80 for younger and p = 0.015 for older; Figure 1). This increase was explained by an expansion of naïve B cells among older subjects to levels greater than those of uninfected controls (p = 0.004; Figure 1). There was a normalization of naïve B cell numbers among younger subjects (p = 0.74; Figure 1). ART was also associated with a normalization of RM cell count in both age-groups (p = 0.69 and p = 0.39 in younger and older subjects, respectively; Figure 1). IT, TLM and MA counts remained similar to controls after 192 weeks of ART in both age-groups. Proportionally RM subset remained lower in the older infected group compared to controls (7% vs. 15%, p = 0.01; Figure 2). After 192 weeks of ART, IT subset contracted in the older infected group to levels below the uninfected controls (2% vs. 8%, p = 0.01). TLM and MA percentages declined to similar levels as controls in both age-groups (Figure 2).

## Discussion

In the present study we sought to further understand the previously observed expansion of peripheral blood B lymphocytes in older subjects treated with ART [10]. Peripheral blood B lymphocyte count increased with ART in both age groups. The supernormal increase in B cells in the older group was a direct result of the striking expansion of the naive B cell subset. The older untreated HIV-infected group, which were with lower peripheral blood RM cells compared to their age-matched controls, experienced normalization of this subset after ART. Regarding B cell phenotypes and vaccine responses, we found an association between RM cell counts and recall immune response to the tetanus vaccine, and a trend towards the same for hepA vaccine in HIV infected subjects, but not controls. Studies have demonstrated that ART leads to near normalization in the peripheral blood B cell profile [1], [4]. However the combined effect of aging and HIV on B lymphocytes is not well understood. We found that after treatment with ART, disturbances seen at baseline return to normal in younger individuals, while older subjects continue to exhibit unexpected differences compared to uninfected age matched controls.

The surprising finding in our study is that the peripheral blood naive subpopulation in older subjects expands to levels above and beyond what is seen in age-matched uninfected subjects. This is especially counterintuitive considering an age-related decline in naive B cell numbers in virally uninfected subjects has been reported [12], [13]. Additionally, we have published data showing older HIV-infected subjects had significantly fewer naïve T cells compared to healthy age-matched controls after 192 weeks of ART [10], [11]. This divergence of super-normal vs. attenuated immunocyte population change of naïve B cell vs. naïve CD4 cell populations during immune reconstitution in older HIV infected individuals is striking. A number of reasons, all of which are speculative, may explain this naïve B cell expansion. It is possible that there is an enhanced capacity of the bone marrow for lymphopoiesis in response to treatment of HIV infection in older individuals. However, decline in bone marrow proliferation potential with age is well documented and there is no evidence to suggest that ART may have an age-differentiated effect on marrow potential [14]. In fact, there is some evidence to suggest that there is decreased bone marrow output as measured by K-deleting recombination excision circles (KRECs) despite prolonged ART [15]. There is evidence to suggest that increased homeostatic proliferation of naïve B cells occurs in response to total B cell deficit [16], though increase to levels higher than normal would not be expected. There may also be a component of altered differentiation in older individuals. We did in fact observe an age-associated contraction in the percentages of IT after ART, though whether this is related to or responsible for naïve B cell expansion is unclear. There has been description of a phenotypically and functionally distinct, late transitional B cell subset that is uniquely sensitive to homeostatic signals and can selectively expand in response to environmental stimuli [17]. Since we did not specifically look for this population we cannot make any conclusions regarding this as possible mechanism for peripheral naïve B cell expansion. Lastly, it is possible that there is an age-associated anatomic redistribution of naïve B cells from lymphoid tissues to peripheral blood in response to ART. In fact, data in Simian Immunodeficiency Virus-Infected Rhesus Macaques suggests that not only does the composition of B cell subsets differ significantly in lymphoid tissues compared to peripheral blood, but that there is also a dynamic exchange between compartments in response to ART [18]. Certainly, one limitation of our study is that we are sampling only peripheral blood and not lymphoid tissues, so we cannot directly address the latter possibility. Due to the limited sample availability, testing these hypotheses was beyond the scope of the present study, and will be the focus of future studies.

Another notable age-related difference in the present study is the observed significantly lower peripheral blood RM counts in the older untreated group compared to age-matched controls. There was recovery of absolute RM counts among the older subjects after ART even though the overall percentage of the RM component remained lower than normal. Moir et al. have previously reported incomplete recovery of resting memory cells despite ART [1], [4] and have speculated that normalization of the memory B cell compartment may be feasible only in those whose therapy was initiated during the acute or early phase of infection, as suggested by Tetanji et al. [19]. Ruffin et al. [20] and Jiang [21] have reasoned that microbial translocation and inflammation which has been shown to play a role in T cell depletion may also be important factors in the deregulated expression of pathways involved in B cell differentiation. Whether ongoing immune activation is responsible for incomplete restoration of memory B cells remains a speculation.

Restoration of the RM may be especially important when considering a positive association between RM cell count at baseline and recall vaccine response to tetanus. The selectivity of this relationship for HIV infection may provide insight into why HIV-infected individuals have impaired humoral response to tetanus vaccine compared with uninfected individuals [22]–[24]. Additionally, results here may help understand parameters guiding the optimal timing of vaccination during HIV infection. It is possible that the response to tetanus vaccine may not be as robust if it is administered before resting memory B cells have reconstituted in response to ART. This maybe especially important in older adults as they have significantly lower resting memory B cells prior to ART and in fact do show rise in absolute resting memory counts after ART. While the exact timeline for the memory B cell reconstitution is not known and it may be impractical to delay vaccination for 192 weeks after ART, this finding gives us an insight into one aspect of impaired humoral response to tetanus vaccine in HIV, and yet another reason to start antiretroviral therapy. We did observe a trend toward an association between higher responses to hepA vaccine and baseline RM counts which is of unclear significance. Responses to neoantigen vaccines would be expected to correlate with naïve cell numbers, an interaction that we did not observe, though higher resting memory cell numbers may reflect upon a more normal naïve B cell functional state.

The limitations of this study include a small sample size. However, despite the small sample size the data show an age-related difference between naïve B cells in response to ART as well as a correlation between resting memory cell numbers at baseline and response to the tetanus vaccine in HIV-infected adults. While the HIV-infected subjects and uninfected controls were imbalanced in terms of race and sex, the intergroup differences that were observed in this study in CD4 and B cell counts are consistent with previously well-characterized HIV-associated changes. Because of imbalances in race and sex, an additional analysis stratified for sex and race (black vs. not black) was performed and did not significantly alter the results.

In summary, the present study demonstrates the different effects of ART on B lymphocyte phenotypes in two different age groups. Despite experiencing a remarkable rise in the total number of B cells, unlike younger adults, older HIV infected individuals fail to restore a normal B cell phenotype distribution in the setting of ART. While there is normalization of the apoptosis-prone and exhausted subpopulations, there is an unexpected rise in the peripheral blood naive population in the older adults after ART. Analysis of the functionality of these expanded naïve cells should provide valuable insight regarding the consequence. Association between the resting memory component at baseline and vaccine response may have implications on the ideal timing of vaccines requiring recall response.

## Supporting Information

Figure S1 **Flow cytometry gating scheme.** Dot plots of representative subject are shown.
(TIF)
