# Supplementary figures and images for: Older Age Is Associated with Peripheral Blood Expansion of Naïve B Cells in HIV-Infected Subjects on Antiretroviral Therapy
Source: PLoS One. 2014 Sep 10;9(9):e107064. doi: 10.1371/journal.pone.0107064 (PMC4160206; doi:10.1371/journal.pone.0107064)

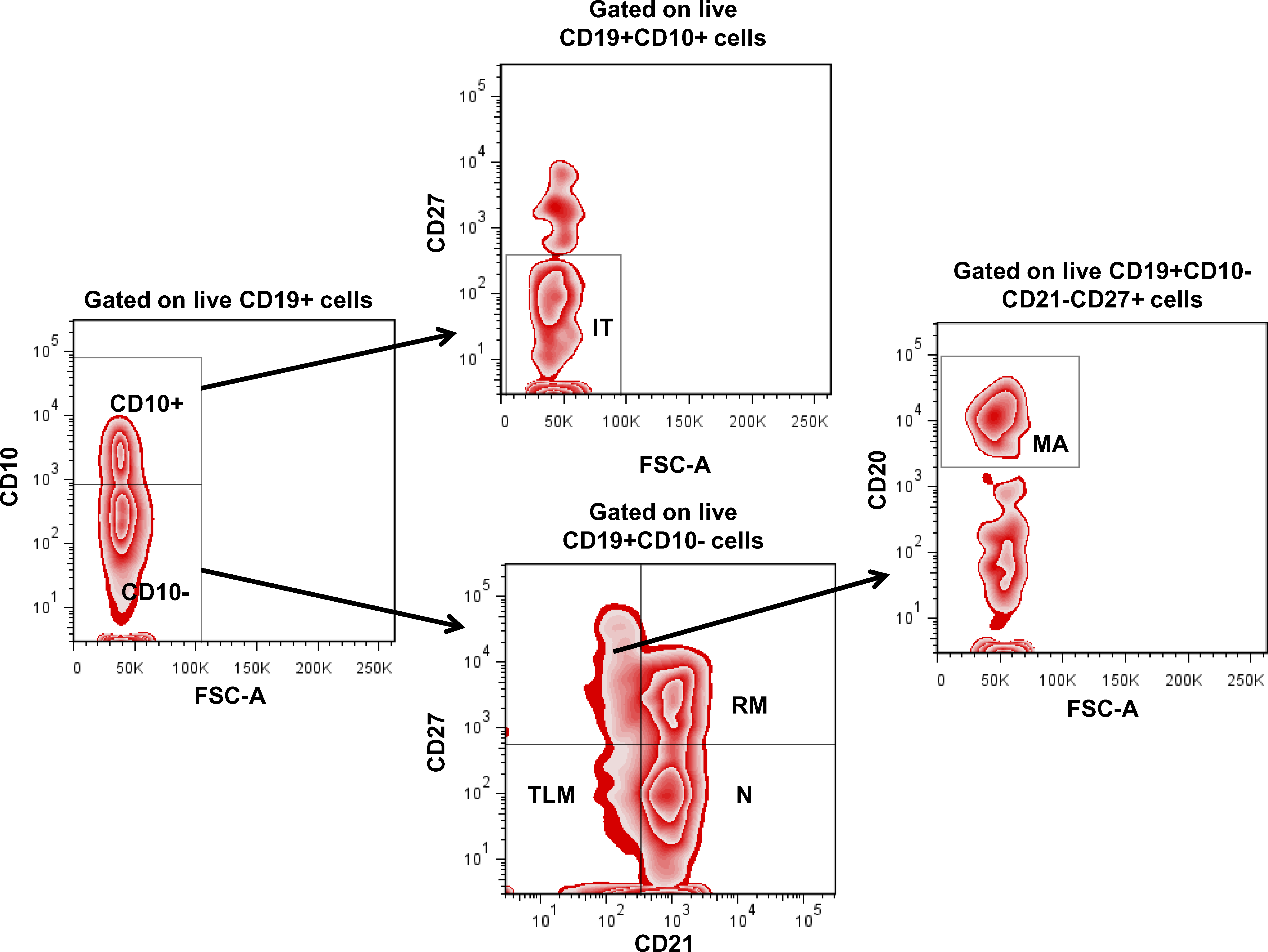

Supplement: Figure S1 — Flow cytometry gating scheme. Dot plots of representative subject are shown. (TIF) [file pone.0107064.s001.tif]
